# TET Methylcytosine Oxidases in T Cell and B Cell Development and Function

**DOI:** 10.3389/fimmu.2017.00220

**Published:** 2017-03-31

**Authors:** Ageliki Tsagaratou, Chan-Wang J. Lio, Xiaojing Yue, Anjana Rao

**Affiliations:** ^1^Department of Signaling and Gene Expression, La Jolla Institute for Allergy and Immunology, La Jolla, CA, USA; ^2^Department of Pharmacology and Moores Cancer Center, University of California at San Diego, La Jolla, CA, USA; ^3^Sanford Consortium for Regenerative Medicine, La Jolla, CA, USA

**Keywords:** TET proteins, 5hmC, T cells, B cells, development, immune gene regulation, chromatin accessibility, cancer

## Abstract

DNA methylation is established by DNA methyltransferases and is a key epigenetic mark. Ten-eleven translocation (TET) proteins are enzymes that oxidize 5-methylcytosine (5mC) to 5-hydroxymethylcytosine (5hmC) and further oxidization products (oxi-mCs), which indirectly promote DNA demethylation. Here, we provide an overview of the effect of TET proteins and altered DNA modification status in T and B cell development and function. We summarize current advances in our understanding of the role of TET proteins and 5hmC in T and B cells in both physiological and pathological contexts. We describe how TET proteins and 5hmC regulate DNA modification, chromatin accessibility, gene expression, and transcriptional networks and discuss potential underlying mechanisms and open questions in the field.

Emerging Themes of TET protein function

1. TET enzymes generate oxi-mC and indirectly mediate DNA demethylation
2. Loss of TET function is associated with lineage dysregulation and cancer
3. Numerous transcription factors bind TET proteins directly
4. TET enzymes facilitate enhancer accessibility and may regulate enhancer function

## Introduction

**Figure 1:**
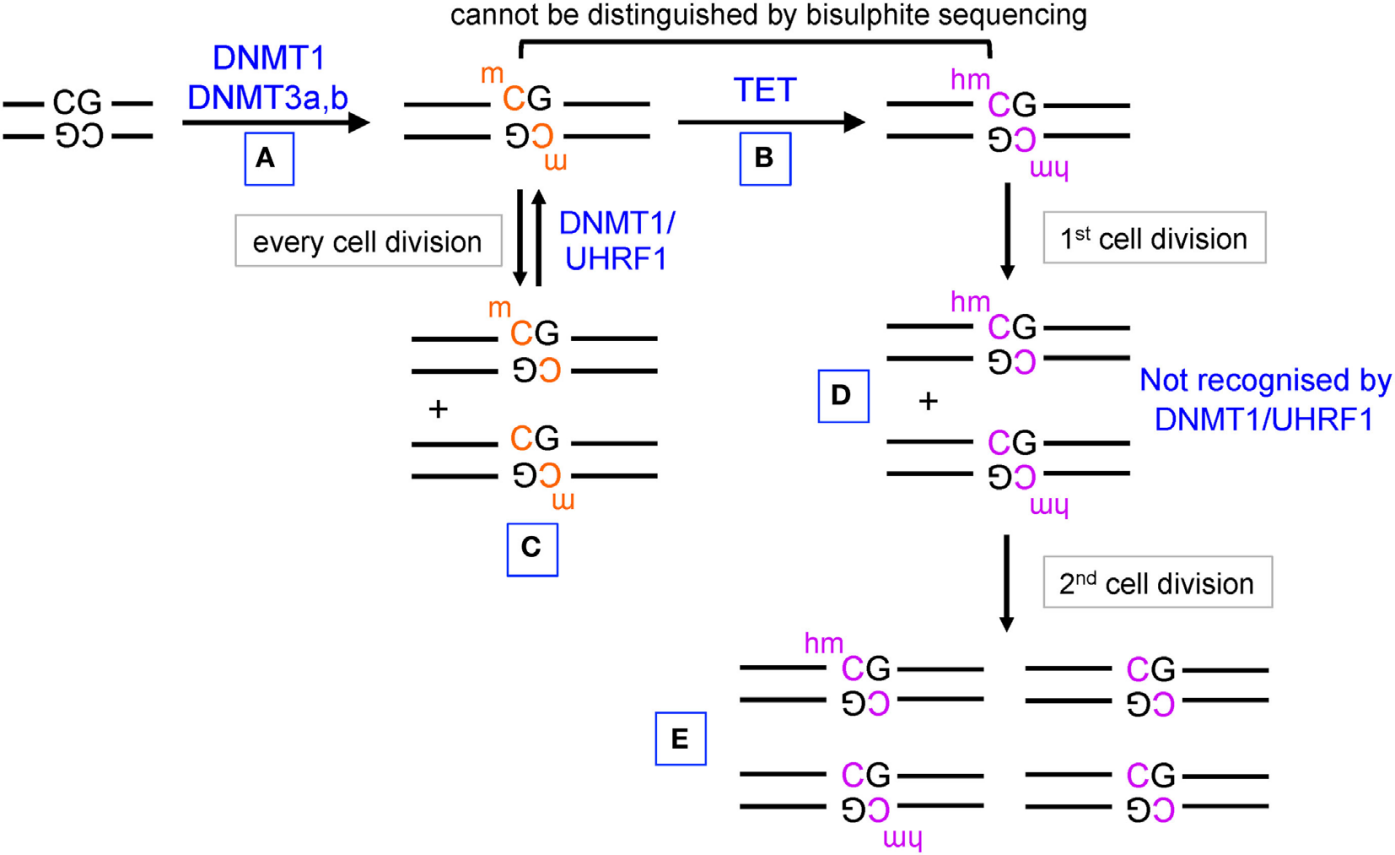
**Pathways of DNA methylation and demethylation mediated by DNA methyltransferase (DNMT) and ten-eleven translocation (TET) proteins**. **(A)** DNMT1, DNMT3a, and DNMT3b establish DNA methylation, primarily at CpG dinucleotides in DNA. **(B)** TET proteins oxidize 5mC to 5hmC. **(C)** During DNA replication, unmodified C is incorporated into the newly synthesized strand of DNA, but the hemimethylated structure is recognized by UHRF1 in the DNMT1/UHRF1 complex and symmetrical CpG methylation is restored by DNMT1. **(D)** 5hmC is also diluted by DNA replication, but hemi-hydroxymethylated CpGs are not recognized by the DNMT1/UHRF1 complex. **(E)** Further rounds of DNA replication result in progressive DNA demethylation.

Until recently, the only known modified base in DNA was 5-methylcytosine (5mC), an epigenetic mark established by the DNA methyltransferases (DNMTs) DNMT1, DNMT3a, and DNMT3b (1) (Figure 1). In mammalian cells, the majority of 5mC is found at symmetrically methylated CpGs (2); DNA replication results in replacement of 5mC on the newly synthesized strand by unmodified C to yield hemimethylated CpGs (1). The “maintenance” DNMT complex, DNMT1–UHRF1, recognizes hemimethylated CpGs and efficiently restores symmetrical CpG methylation at most genomic locations, consistent with the notion that in cells with unimpaired DNMT activity, DNA methylation is generally a stable and heritable epigenetic mark (1) (Figure 1, left). DNA methylation ensures genome stability by suppressing transposon reactivation (1); but the extent to which DNA methylation has a direct causative role in gene regulation is currently controversial (3, 4). Genome-wide bisulfite sequencing in several cell types has established that the promoters of the most highly expressed genes show the lowest levels of CpG methylation and, conversely, that dense CpG methylation of promoters is associated with low gene expression (2, 5, 6). Whether this association implies causality, and if so in which direction, is unclear: there is considerable evidence that DNA methylation follows rather than “instructs” gene expression (3, 4, 7) as further discussed below.

**Figure 2:**
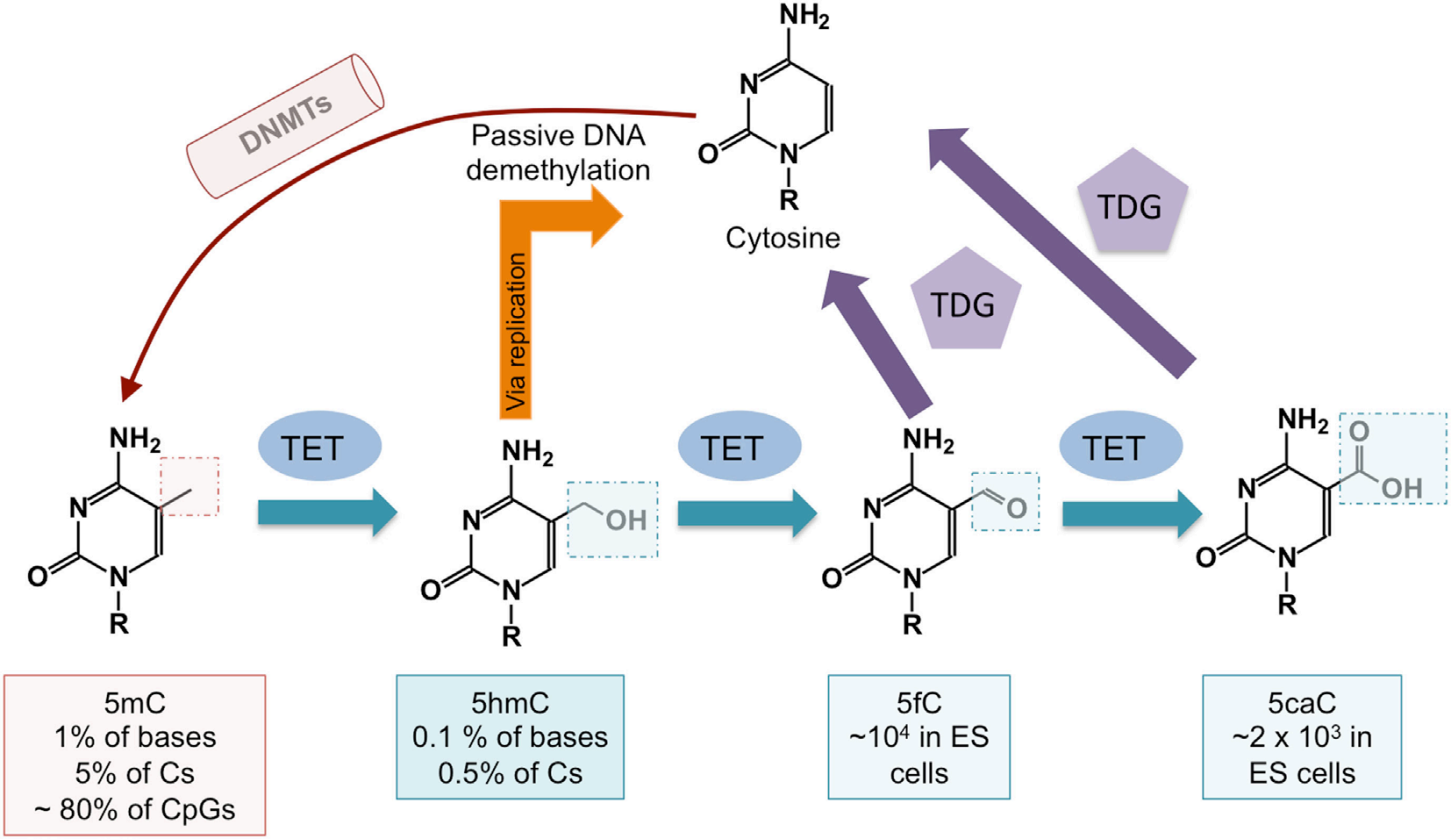
**DNA demethylation pathways controlled by ten-eleven translocation (TET) proteins**. DNA cytosine methylation is established by DNA methyltransferases (DNMTs). TET proteins oxidize 5mC to 5-hydroxymethylcytosine (5hmC), which is a relatively stable modification. 5-methylcytosine (5mC) is passively diluted via DNA replication as cells divide, but symmetrical methylation is restored by the maintenance methyltransferase complex DNMT1/UHRF1. 5hmC is also passively diluted as a function of cell division, and this results in DNA demethylation (see Figure 1). DNA demethylation can also occur via further oxidization of 5hmC by TET proteins to 5-formylcytosine (5fC) and 5-carboxylcytosine (5caC). Both 5fC and 5caC can be excised by thymine DNA glycosylase (TDG), resulting in their replacement with unmodified cytosine through base excision repair.

The discovery that ten-eleven translocation (TET) proteins are 5-methylcytosine oxidases revolutionized our understanding of DNA cytosine modification, by providing plausible biochemical mechanisms for the reversal of DNA methylation (8–11). TET proteins are 2-oxoglutarate- and Fe(II)-dependent dioxygenases that catalyze the oxidation of the methyl group of 5mC to 5hmC as well as the further oxidation products 5-formylcytosine (5fC) and 5-carboxylcytosine (5caC) in DNA (12, 13) (Figure 2). TET enzymes can act as mediators of “active” (replication-independent) DNA demethylation, achieved through excision of 5fC and 5caC by thymine DNA glycosylase (TDG) followed by replacement with an unmethylated cytosine through base excision repair (13–15) (Figures 1 and 2). In most systems that have been investigated so far, however, TDG-mediated 5fC/5caC excision does not seem to be the major route of DNA demethylation: rather, the predominant mechanism involves TET-mediated conversion of 5mC to 5hmC followed by passive replication-dependent dilution of 5hmC (16, 17) (Figures 1 and 2). This process occurs because the DNMT1–UHRF1 complex does not recognize hemi-hydroxymethylated CpGs (10, 18).

To evaluate DNA modification status, most studies have used bisulfite-mediated deamination of unmodified C to uracil, which is read as T after PCR amplification (19). This method does not distinguish 5mC from 5hmC (20), nor does it discriminate among unmodified C, 5fC, and 5caC (10, 21, 22). Despite the fact that 5hmC comprises only a small fraction of 5mC (Figure 2), it is regulated in a considerably more dynamic fashion (23). In all future analyses, it will be necessary to specify DNA modification status (5mC, 5hmC, or 5mC + 5hmC if not distinguished), and to employ methods such as oxidative bisulfite sequencing (24) or TAB-seq (25) [reviewed in Ref. (10, 22)] that can quantitatively measure both 5mC and 5hmC (26–28).

## General Features of TET Proteins and 5hmC

### The Mammalian TET Proteins

**Figure 3:**
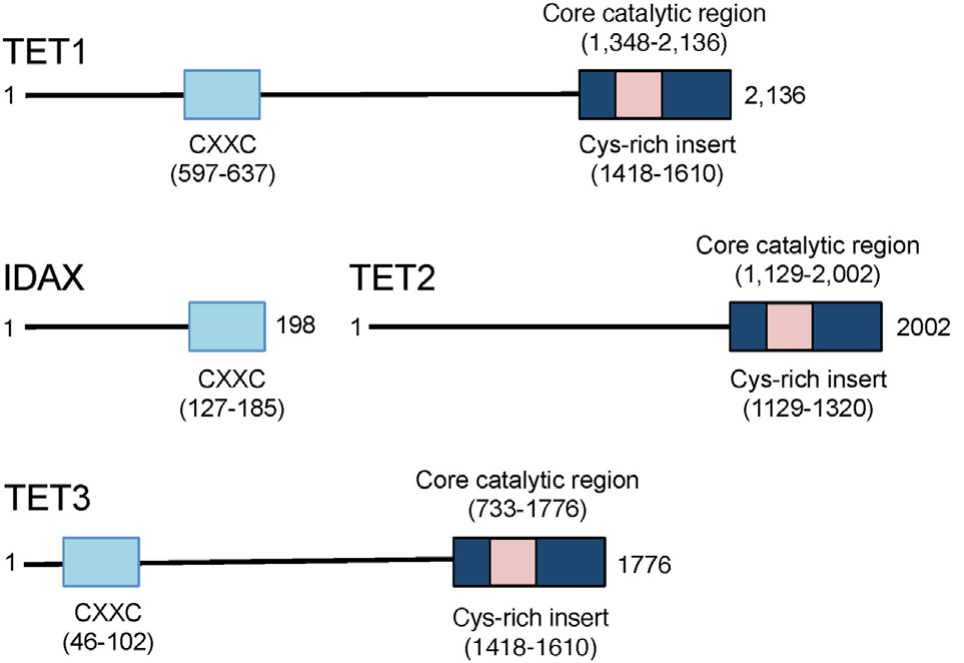
**Schematic representation of the domain structure of the three mammalian ten-eleven translocation (TET) proteins TET1, TET2, and TET3**. TET1 and TET3 possess an N-terminal CXXC domain that recognizes unmethylated CpGs in DNA. The CXXC domain of the primordial *TET2* gene became separated from the catalytic domain due to chromosomal inversion during evolution, and the CXXC domain evolved to become a separate protein, CXXC4 or IDAX. The catalytic regions of all three TET proteins are located at the C-terminus.

Representatives of the TET/JBP superfamily exist in every metazoan organism using DNA methylation, consistent with a conserved role in the regulation of DNA methylation (29). The three mammalian TET proteins, TET1, TET2, and TET3 (Figure 3), arose from a common ancestral gene that underwent triplication in jawed vertebrates (10, 29, 30). TET1 and TET3 both possess an N-terminal CXXC DNA binding domain, which recognizes unmethylated CpGs (29), but TET2 has lost its CXXC domain due to a chromosomal inversion, resulting in the formation of a separate gene known as CXXC4 or IDAX (8, 29, 30) (Figure 3). TET1 is highly expressed in embryonic stem (ES) cells and its expression drops following differentiation of ES cells to embryoid bodies (9, 31). TET1 is also highly expressed in primordial germ cells (16, 32). TET2 is expressed at lower levels than TET1 in ES cells and its expression first drops and then increases upon differentiation; it is expressed in numerous differentiated organs and cell types in the adult (10, 11). TET3 is highly expressed in oocytes and zygotes (33), and loss of TET3 in mice results in perinatal lethality (10, 11).

### Levels and Distribution of oxi-mC

TET function in cells can be assessed by measuring 5hmC levels in nuclear DNA, using mass spectrometry or DNA dot blot when cell populations are involved, or by immunocytochemistry or flow cytometry at the single-cell level. 5hmC is present at higher levels in neurons than in other cell types (34) and is abundant in Purkinje neurons where it comprises ~40% of the level of 5mC (35). ES cells also have high levels of 5hmC, varying between 5 and 10% of the levels of 5mC. In contrast, 5hmC is present at only 1% of the total level of 5mC in some immune populations (36). 5fC and 5caC are even less abundant, regardless of cell type (12). Notably, 5hmC as well as the less abundant 5fC are stable epigenetic marks, and all three oxi-mC bases are thought to be capable of recruiting specific readers (37–39). The biological importance of oxi-mC recognition by these putative readers has not yet been explored.

Enrichment of 5hmC is observed in the gene body of very highly expressed genes as well as in active enhancers in ES cells (40–42), neural cells (37), hematopoietic stem/progenitor cells (HSPCs) (43), T cells (44, 45), and B cells (46). Notably, 5hmC is depleted from the transcription start site (TSS) of highly transcribed genes. Consistent with its deposition in the gene body, the genome-wide distribution of 5hmC correlates with that of histone 3 lysine 36 trimethylation (H3K36me3), and polymerase II (Pol II) in thymocytes, emphasizing its positive correlation with transcriptional elongation (44).

### Recruitment of TET Proteins to DNA

Ten-eleven translocation proteins are likely to be recruited to the DNA by transcription factors. Recent research in ES cells suggested that TET1 is initially recruited to the DNA then interacts with SALL4A, which subsequently recruits TET2; it is TET2 in this context that is suggested to play the dominant oxi-mC-producing role (47). Whether other synergistic interactions like this one take place in immune cells remains to be shown. Moreover, PU.1 has been shown to interact with TET2 and can bind genes that undergo DNA demethylation (48). EBF1 can also interact with and thus recruit TET2 to specific loci (49). Another transcription factor, WT1, can interact with TET2 and mediate the recruitment of TET2 to genes resulting in their upregulated expression (50). In addition to these interactions of TET proteins with transcription factors that positively regulate gene expression, TET2 is reported to interact with IkBζ, which recruits it to the IL-6 locus (51); TET2 in turn interacts with and recruits HDAC2 mediating the repression of IL-6 (51).

### 5hmC Levels Correlate Positively with Gene Expression

The emergence of genome-wide methods to map 5hmC (Box 1) has allowed the assessment of 5hmC distribution during T cell development and lineage specification (44), as well as during *in vitro* polarization of naïve CD4^+^ T cells toward T helper subsets and induced T regulatory cells (iTregs) (44, 45, 52). It is clear from these and other studies that 5hmC is enriched in the gene bodies of very highly expressed genes as well as at the most active enhancers (44, 45). Once again, the direction of causality is not clear: 5hmC may travel with the SET methyltransferase complex (53) and thereby with RNA Pol II, thus passively depositing 5hmC at transcribed regions. Alternatively, its presence in transcribed regions may facilitate Pol II elongation. These possibilities are not mutually exclusive; one can envision a positive feedback loop where initial transcription through the locus allows 5hmC deposition by TET proteins, after which the deposited 5hmC increases chromatin accessibility (see below), thus facilitating subsequent cycles of Pol II-mediated transcription. To distinguish these possibilities, it will be necessary to perform kinetic analyses comparing the rate of change in 5hmC levels and in gene transcription following acute deletion of one or more TET genes, as well as after restoration of catalytically active and inactive TET proteins. Biochemical experiments quantifying *in vitro* transcription on chromatinized DNA templates will also be needed to pin down how 5hmC in gene bodies affects Pol II-mediated transcriptional initiation and elongation.

Box 1 **Techniques for 5hmC analysis.**

**Low throughput:** restriction enzyme and PCR

**Sequencing based:**

1. Enrichment-based:
  i hMeDIP (54)
  ii CMS-IP (40, 55)
  iii T4-BGT-based enrichment
    GLIB (40, 56)
    hMeSeal (57)
  iv JBP1-seq (58)
2. Base resolution
  i oxidative bisulfide sequencing (oxBS) (24)
  ii RRHP (59)
  iii PvuRts1I (60)
  iv Aba-seq (61)
  v TAB-seq (25)
  vi HELP-GT (62)
  vii SMRT (63)
3. Other variations
  i Low input/single cell (64, 65)
  ii oxBS-array (66)

### 5hmC Distribution Corrselates with Chromatin Accessibility

Studies of TET-deficient mice have also revealed a strong association of 5hmC distribution with accessible regions in chromatin. Among regions identified as differentially accessible in wild type (WT) versus Tet2/3-deficient (*Tet2/3 DKO*) invariant NKT (iNKT) cells (67) and B cells (46), the regions that lost accessibility in *DKO* cells compared to WT were those enriched for 5hmC in WT cells. These results suggest a regulatory role of TET proteins in maintaining chromatin accessibility and thus allowing the recruitment of transcription factors that can execute lineage-specific transcription programs. Once again, the direction of causality needs to be worked out: is 5hmC deposited at accessible regions because TET is recruited to those regions by transcription factors, or does the presence of 5hmC intrinsically increase chromatin accessibility? Again, these possibilities are not mutually exclusive: kinetic and biochemical analyses, asking whether changes in chromatin accessibility occur after acute ablation or restoration of TET function, will be necessary to establish possible causal relationships by defining the kinetic sequence of events.

### The Effects of TET Loss-of-Function Are Most Apparent in Rapidly Proliferating Cells

In both myeloid-lineage precursors (68) and during lymphoid differentiation (46, 67), the most striking consequences of TET loss-of-function are observed in cells undergoing rapid proliferation. This has also been observed for other epigenetic marks such as H3K27me3, the product of the PRC2 complex (69, 70). The simplest explanation is that these epigenetic marks are stable and are primarily lost as a consequence of DNA replication, as formally established for 5hmC (18, 71). Under these conditions, inactivation of proteins that control the generation or deposition of these epigenetic marks is not sufficient to erase the marks from the genome. Rather their functions are revealed through passive dilution of the marks during subsequent waves of proliferation, especially in the absence of proteins that can reestablish them *de novo* at the genomic loci that they control.

### TET Loss-of-Function and the Dysregulation of Cell Lineage Specification Programmes

As expected from the positive correlation of 5hmC levels with gene expression, 5hmC is enriched in the gene bodies of key lineage-specifying factors—including Th-POK, T-bet, Runx3, Gata3, RORγt, Foxp3, and Bcl6 (45, 52)—in the cell types and at the developmental stages where these factors are most highly expressed (44). Similarly, 5hmC is enriched in the transcribed regions of genes encoding key cytokines important for immune responses, such as IL-4, IFNγ, and IL-17, specifically in the T cell subsets that secrete these cytokines (45). Similar findings were reported in human CD4 T cells during their differentiation (72, 73). Moreover, 5hmC-enriched regions in human CD4 T cells are significantly enriched for genetic variants associated with T cell diseases such as diabetes and multiple sclerosis, as well as with regions involved in T cell-specific chromosomal interactions (72). 5hmC deposition is also associated with demethylation of the *Cd4* locus (74) as well as the *Pdcd1* (encoding PD-1) promoter (75).

**Figure 4:**
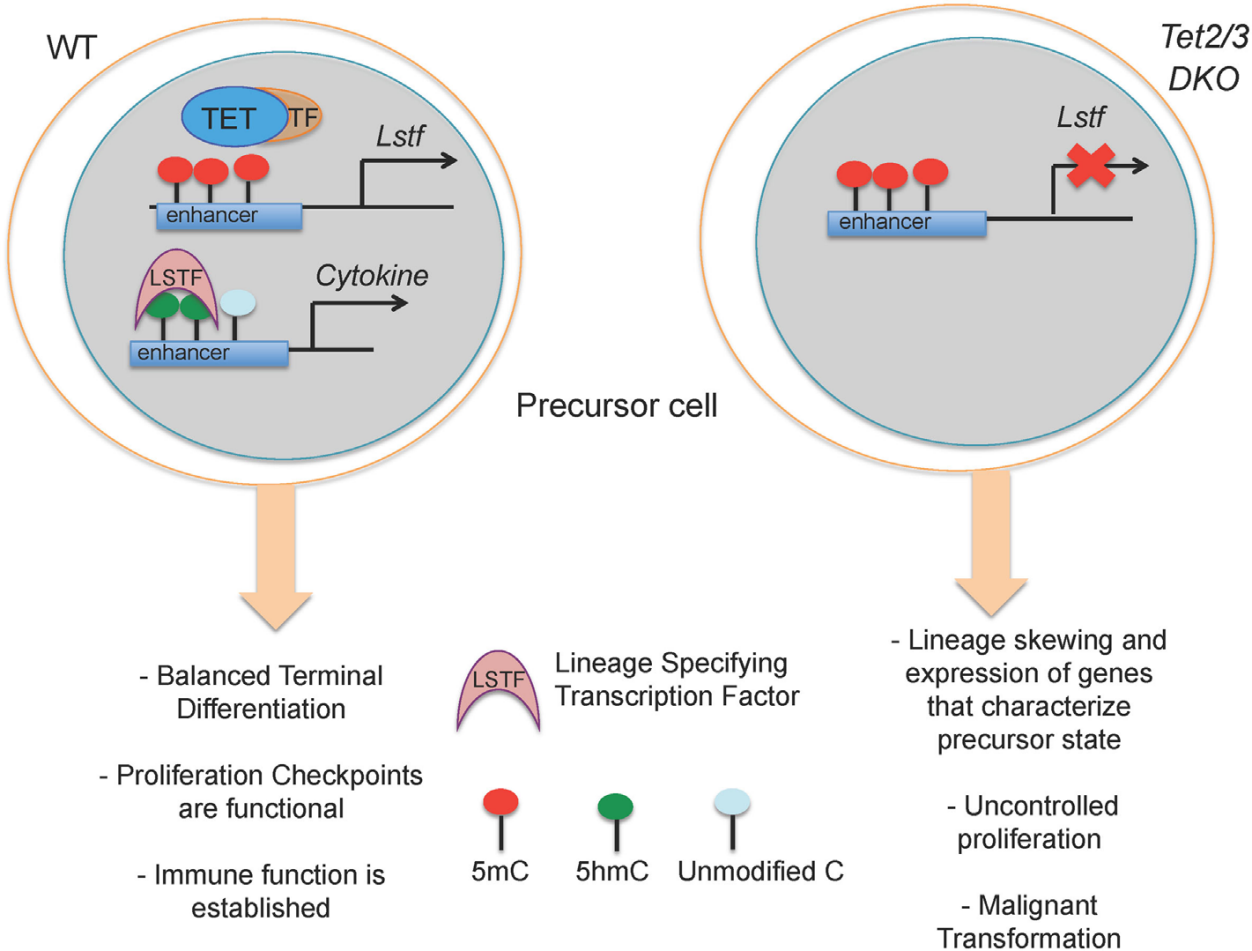
**Suggested model for how loss of TET2 and TET3 affects immune cell biology**. (Left) In a wild-type precursor cell, TET2 and TET3 facilitate DNA demethylation and support the execution of a cell-specific gene expression program resulting in correct lineage specification and controlled cell proliferation. (Right) In *Tet2/3* DKO precursor cells, the gene expression program is profoundly altered with consequent lineage skewing, aberrant proliferation and malignant transformation.

**Figure 5:**
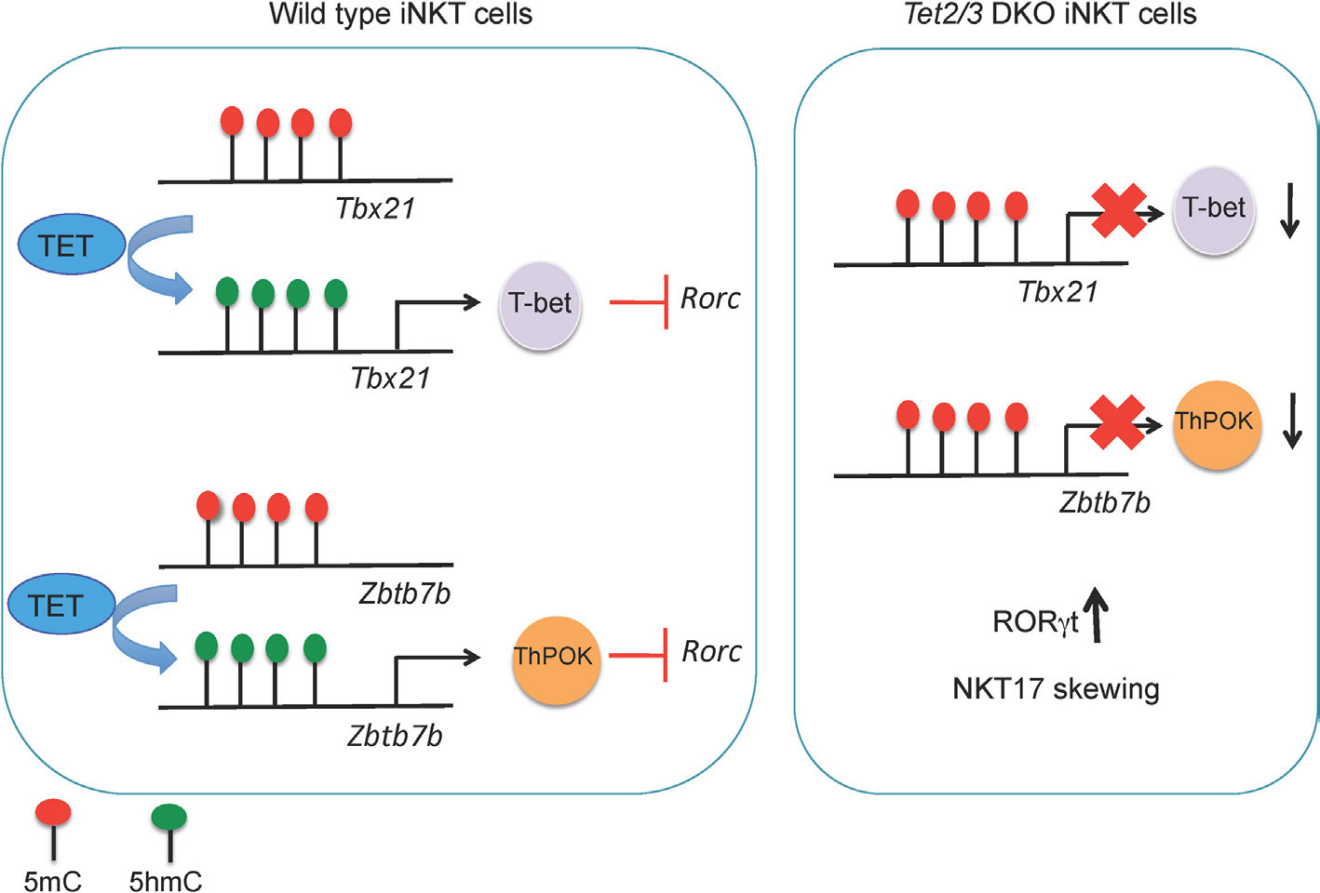
**(Left) TET2 and TET3 demethylate the *Tbx21* and *Zbtb7b* loci, a process associated with increased expression of T-bet and ThPOK, respectively, in wild-type invariant NKT (iNKT) precursor cells**. T-bet and ThPOK suppress *Rorc* (encoding RORγt) expression. (Right) in *Tet2/3* DKO iNKT precursor cells, the *Tbx21* and *Zbtb7b* loci remain methylated and Tbet and ThPOK are expressed at low levels. As a result, RORγt is expressed and *Tet2/3* DKO iNKT cells are skewed toward the NKT17 lineage.

**Figure 6:**
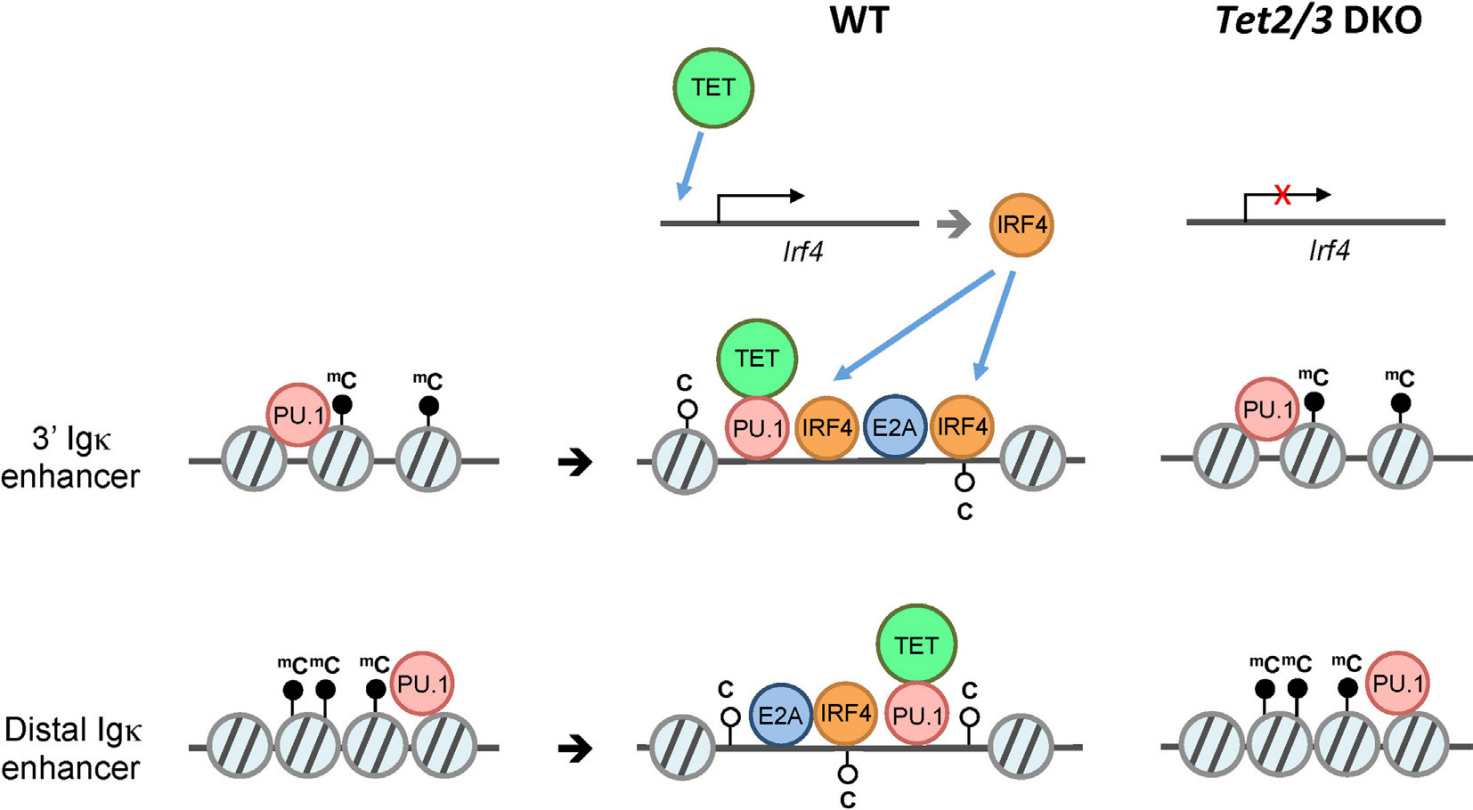
**Model for ten-eleven translocation (TET)-mediated regulation of Igκ enhancers**. B cells undergo V(D)J rearrangement of immunoglobulin (Ig) to diversify the B cell receptor repertoire during development. While Ig heavy chain genes (IgH) are rearranged primarily at the pro-B cell stage, the Ig light chain genes Igκ and Igλ are rearranged at around the pre-B cell stage. Prior to Igκ and Igλ rearrangement, the pioneer transcription factor PU.1 associates with numerous enhancers including the 3′ and distal Igκ enhancers despite a “closed” chromatin state (left). Subsequently, PU.1, and potentially E2A recruit Tet2 and Tet3, which facilitate 5hmC deposition and/or DNA demethylation with an associated increase in chromatin accessibility of the enhancers, thus presumably facilitating the binding of additional transcription factors (middle). In the absence of Tet2 and Tet3 (right), the enhancers show increased DNA methylation and decreased chromatin accessibility compared to their wild-type counterparts. In addition to directly regulating Igκ enhancers, TET2/3 regulate the expression of IRF4 and IRF8 (not shown), two transcription factors that are important for Igκ rearrangement (top).

These correlations explain the critical roles of TET proteins in the regulation of developmental choices in both T and B cell lineages (46, 67, 76) (Figure 4). As discussed below, we have used mice lacking Tet2 and Tet3, the two TET proteins most highly expressed in differentiated cells, to examine the role of profound TET loss-of-function in T and B cell subsets. Consistent with passive replication-dependent dilution of 5hmC, lineage skewing is most apparent at and after developmental stages characterized by rapid proliferation, when 5hmC is most efficiently diluted. At such developmental stages, TET proteins regulate the expression of genes encoding key transcription factors that shape the fate of the cells in which they are expressed, including T-bet and ThPOK in T cells and IRF4/8 in B cells (46, 67) (Figures 5 and 6). By affecting the expression levels of these factors, loss of TET function also indirectly affects the regulatory networks in which these factors participate, resulting in amplification of the effects and further deregulation of cell type-specific gene expression programs.

Lack of Tet2 and Tet3 results in entrapment of T and B cells in an immature state characterized by aberrant expression of genes that control proliferation and defective maturation. In these *Tet2/3 DKO* mice, the hyperproliferation of immature cell types results in malignant transformation and the emergence of cancers, as discussed in more detail below (Figure 5).

## Role of DNMTs in T and B Cells

### T Cells

The role of DNMTs in T cells has been extensively investigated. Cre-mediated deletion of *Dnmt1* in double-negative (CD4^–^CD8^–^, DN) thymocytes using *LckCre* revealed a role for Dnmt1 in survival of αβ T cells as well as lineage specification of γδ T cells during thymic development, whereas deletion at the double-positive (CD4^+^ CD8^+^, DP) stage using *CD4Cre* resulted in decreased proliferation but increased cytokine production by naïve T cells (77). Cre-mediated deletion of *Dnmt1* in activated CD8^+^ T cells using Granzyme B-Cre resulted in decreased expansion of antigen-specific CD8 T cells upon viral infection and moderately affected their differentiation to effector and memory CD8 T cells (78). Loss of Dnmt3a in CD4^+^ T cells resulted in a slight increase in ectopic cytokine expression and lineage plasticity (79). Moreover, T cell-specific deletion of *Dnmt3a* resulted in skewing of CD8 T cells toward memory precursors and a decrease of effector CD8^+^ subsets upon viral infection (80). In all cases, there is a strong negative correlation of DNA methylation (5mC) at promoters with gene expression. For instance, *Cd4* is sequentially turned on and off in thymocytes depending on the developmental stage, in a manner that is likely to be epigenetically controlled (74, 81). Similarly, as Lck transcription is upregulated, CpGs in exon 1 through intron 2 become progressively demethylated (82). Likewise, demethylation within the transcribed sequences of *Il4* and *Ifng* correlates with high expression levels of these cytokines in Th2 and Th1 cells, respectively (83).

Notably, detailed analyses of DNA demethylation support the notion that DNA demethylation follows gene transcription. For instance, the *Il4* promoter was shown to be demethylated in naïve T cells and to remain demethylated in Th1 cells, and there was no additional demethylation during the initial few days of Th2 differentiation, despite the emergence of fully differentiated Th2 cells capable of producing IL-4 (84). In contrast, in Th2 cells that had been maintained for many weeks, demethylation extended from the IL-4 promoter deeply into the gene body and the cells produced high levels of IL-4 (84). Similar conclusions can be drawn from a study of two conserved regions in the *Pdcd1* locus, which encodes the inhibitory receptor PD-1 (85). Both regions were methylated in naïve CD8^+^ cells, where the *Pdcd1* gene is not expressed; upon acute viral infection, both regions transiently lost methylation in effector CD8^+^ cells that express PD-1 and partially regained methylation when PD-1 was silenced in memory CD8^+^ T cells (85). Both regions also remained unmethylated in PD-1-expressing “exhausted” CD8^+^ T cells that are unresponsive to further stimulation with antigen (85); these cells emerge during chronic viral infection as the result of a negative feedback mechanism stemming from prolonged T cell receptor (TCR) stimulation (86). However, the levels of DNA demethylation did not correlate quantitatively with gene expression, since equivalent demethylation was observed in exhausted cells in which PD-1 expression spanned a relatively wide range (85). Together these studies indicate that the extent of DNA demethylation correlates qualitatively, but not kinetically or quantitatively, with the level of gene expression, supporting the hypothesis that DNA demethylation does not “instruct” gene transcription (3, 4).

With the advent of high-throughput sequencing, many groups have obtained genome-wide DNA methylation maps for various mouse and human T cell types. Analysis of changes in DNA methylation during human thymic development using Infinium Human Methylation 450 Bead Chips revealed that DNA demethylation was more frequent than *de novo* DNA methylation, and DNA demethylation more strongly correlated with gain of gene expression (87). Moreover, immunoprecipitation of 5mC-enriched DNA fragments followed by genome-wide sequencing (MEDIP-seq) of naïve mouse CD8 cells and CD8 effector T cells at day 8 post acute lymphocytic choriomeningitis virus infection showed that gain of DNA methylation in promoter regions correlated negatively with gene expression (88).

### T Regulatory Cells (Tregs)

The transcription factor Foxp3 is essential for the development and function of Tregs (89–91). Foxp3 expression during Treg cell differentiation is regulated by three conserved non-coding sequence (CNS) elements located at the Foxp3 gene locus (92–94). Of these, *CNS2* was originally identified as a Treg-specific demethylated region (TSDR) (95) that is fully methylated in naïve T cells and differentiated T cell subsets, but demethylated in Tregs. An unusual feature of *CNS2* is that its methylation status regulates the stability of Foxp3 expression rather than the initial level of expression (92–94). A few more TSDRs were also reported within genes that are important for Treg cell differentiation and function, such as *Ctla4, Il2ra (Cd25), Ikzf4*, and *Tnfrs18* (96).

Early findings emphasized the role of DNA methylation in the control of Foxp3 expression. Inhibition of DNA methylation by the DNMT inhibitor 5-azacytidine (97, 98), or genetic deletion of the gene encoding Dnmt1 in mice (99), eliminated the requirement for TGFβ for the generation of iTregs and promoted Foxp3 expression in thymic and peripheral Foxp3-negative T cell populations in response to TCR stimulation alone. At least two methyl-binding proteins, Mbd2 and MeCP2, have been reported to have a role in maintaining Foxp3 expression and Treg suppressive function (100, 101). Both proteins bind CNS2 but their functions appear to be different: deficiency of Mbd2 results, somewhat paradoxically, in increased methylation of *CNS2* (100) whereas deficiency of MeCP2 was reported to result in decreased histone 3 (H3) acetylation (101). In a subsequent section, we discuss the role of TET proteins in regulating the methylation status of *CNS1, CNS2*, and other TSDRs (26, 102, 103).

### B Cells

B cells undergo dynamic changes in DNA modification status during their development, with an estimated 30% of all CpGs exhibiting changes at distinct genomic regions depending on developmental stage (104). Ablation of the gene encoding the maintenance methyltransferase Dnmt1 completely halted early B cell development (105), but the *de novo* methyltransferases Dnmt3a and Dnmt3b were dispensable for B cell development in conditional *Mb1Cre* Dnmt3a/b-deficient mice, although the B cell receptor repertoire was skewed toward increased usage of proximal Vκ genes (106). Thus, maintenance of global DNA methylation is essential for B cell development, while *de novo* methylation is important for proper immunoglobulin (Ig) gene rearrangement. Moreover, during their development in the bone marrow, B cells selectively express and rearrange only one of the two alleles encoding Ig heavy and light chains, respectively, a process termed “allelic exclusion.” Monoallelic expression of the Igκ chain correlates with preferential demethylation of the rearranged allele (107).

After antigen stimulation and T cell help, naïve B cells become germinal center (GC) B cells. In contrast to bone marrow B cells, GC B cells undergo global DNA demethylation mostly at heterochromatic regions and re-methylation at polycomb-repressed regions marked by histone 3 lysine 27 (H3K27) methylation in humans (104). However, consistent with increased expression of Dnmt1 during GC B cell differentiation, hypomorphic Dnmt1 mice failed to generate GCs, suggesting that maintenance of a specific DNA methylation pattern is critical (108). Similarly, differentiation from naïve B cells to plasma cells is also accompanied by large scale DNA demethylation, with around 10% of CpGs exhibiting significant changes (109), and inhibition of DNA methylation by 5-azacytidine increased the percentage of plasma cells in a manner dependent on cell division. DNA demethylation at these regions correlated with binding of transcription factors NFκB and AP-1 at early stages of B to plasma cell differentiation and binding of IRF and Oct-2 at later stages, suggesting that demethylation was secondarily mediated through transcription factor binding, possibly through recruitment of TET proteins as further discussed below.

## TET Proteins Affect Multiple Aspects of Hematopoietic and Immune Cell Development and Function

The effects of individual and combined *TET* gene deletions in immune/hematopoietic cell populations have been assessed in several studies. The overall conclusion is that *combined deletion of at least two TET genes is required for profound phenotypic effects*. Mice lacking individual TET enzymes (Tet1, Tet2, or Tet3) display mild overall phenotypes that in most cases only become apparent after many months to more than one year. In contrast, double deletion of Tet2 and Tet3, the two TET proteins most highly expressed in differentiated tissues, has far more deleterious effects, which often become obvious within a few weeks.

### Myeloid-Lineage Cells

Tet2-deficient macrophages and dendritic cells produce more IL-6 in response to stimulation, rendering Tet2-deficient mice more susceptible than WT mice to endotoxin-induced shock and DSS-induced colitis (51). However, most studies on Tet2 loss-of-function mutations have focused on the tumor suppressor function of Tet2. Tet2-deficient mice show increased numbers of hematopoietic stem/progenitor cells (HSPCs) and increased self-renewal *in vitro* (110). Some Tet2-deficient strains display progressive defects in myelopoiesis over a time course of 20 weeks, culminating in a myeloid neoplasia reminiscent of human CMML (chronic myelomonocytic leukemia) (111). Tet3-deficient mice show a mild dysregulation of HSPC proliferation, but they do not develop leukemia, except occasionally with low penetrance and very long latency (112). In contrast, acute inducible deletion of both Tet2 and Tet3 in hematopoietic stem cells resulted in the rapid emergence of an aggressive myeloid leukemia with 100% penetrance in only 4 weeks (68). Curiously, however, mice lacking Tet1 alone, or both Tet1 and Tet2, developed B cell rather than myeloid leukemias with relatively long latency (12–15 months) (113). Thus, Tet1 loss-of-function predisposes to B cell malignancies with long latency, whereas Tet2 and Tet3 act together to suppress myeloid leukemogenesis. Plausible scenarios are that Tet1 is poorly expressed in differentiated cells (21), is required for the progression of myeloid leukemia, or both. Another possible explanation is that Tet1 and Tet2 have distinct functions, whereas Tet2 and Tet3 have at least partially overlapping roles. Indeed, Tet1 has a major role in 5hmC deposition at promoter/TSS regions in mouse embryonic stem (mES) cells, whereas Tet2 and Tet3 seem to act predominantly at distal enhancers (44, 46, 67, 76, 114).

### T Cells

Tet2 modulates cytokine gene expression during CD4^+^ T cell differentiation, and the observed phenotypes correlate with aberrant modification (impaired demethylation) of known or putative enhancer elements in genes encoding relevant cytokines and transcription factors (45, 102). Deletion of both Tet2 and Tet3 in T cells (26) resulted in a dramatic lymphoproliferative disease that was lethal by 8 weeks (67); the causes included the uncontrolled, antigen-driven expansion of a normally minor subpopulation of T cells known as iNKT cells (67), as well as impaired Treg function due to unstable Foxp3 expression (26). These phenotypes were detectable, but much less apparent, in mice with individual loss of Tet2 or Tet3 alone (67).

Invariant NKT cells are selected in the thymus by recognition of lipids through antigen presentation via CD1d, an MHC I-like molecule (115, 116). iNKT cells can potently secrete cytokines and can act as first responders in infections. They can be classified based on the expression of key lineage-specifying transcription factors: NKT1, NKT2, and NKT17 cells express T-bet, Gata3, and RORγt, respectively (117, 118). An alternative classification of iNKT cells is based on expression of surface markers (115): stage 0 precursors are CD24^+^ and do not express CD44 or NK1.1; stage 1 iNKT cells downregulate CD24 and are CD44^−^NK1.1^−^; stage 2 iNKT cells are CD44^+^ NK1.1^–^; and stage 3 cells upregulate NK1.1 to become CD44^+^NK1.1^+^. iNKT cell subsets are heterogeneous and transcriptionally very different, reflecting their distinct effector functions (119–121).

Tet2 and Tet3 act together to control iNKT cell expansion and cell lineage specification (67). *Tet2/3* T-DKO mice showed an impressive expansion of iNKT cells even at very young ages. The increased absolute numbers of iNKT cells in *Tet2/3 DKO* mice and the concomitant increase of IL-4 secretion gave rise to innate-like CD8 T cells (67). Further analysis revealed skewing of the *Tet2/3* DKO iNKT cells toward the NKT17 lineage, as judged by upregulation of RORγt and increased secretion of IL-17 (67). In cells lacking Tet2 and Tet3, the genes encoding Tbet and ThPOK did not gain 5hmC, remained methylated, and did not achieve the high expression levels observed in WT counterparts (67) (Figure 4). This resulted in higher expression of RORγt and skewing of the *Tet2/3* DKO iNKT cells to the NKT17 lineage, whereas NKT2 and NKT1 lineages were underrepresented (67). Examination of iNKT subsets (117, 119)—stage 0 precursors, NK1.1^−^ and NKT1^+^ subsets—demonstrated that concomitant loss of *Tet2* and *Tet3* resulted not only in altered numbers of iNKT cells but also profoundly influenced gene expression programs and thus the identity and function of each subset (67). NKT1 cells were not only fewer in number, but their identity was greatly altered. In addition to their effector function, *Tet2/3* DKO iNKT cells also exhibit increased proliferative capacity, a property that can explain their *in vivo* expansion (67), associated with upregulation of genes such as Myc and Lef1 that control iNKT cell proliferation in NKT2, NKT17, and even in NKT1 cells where Lef1 expression should have been turned off (119, 120, 122).

### T Regulatory Cells

**Figure 7:**
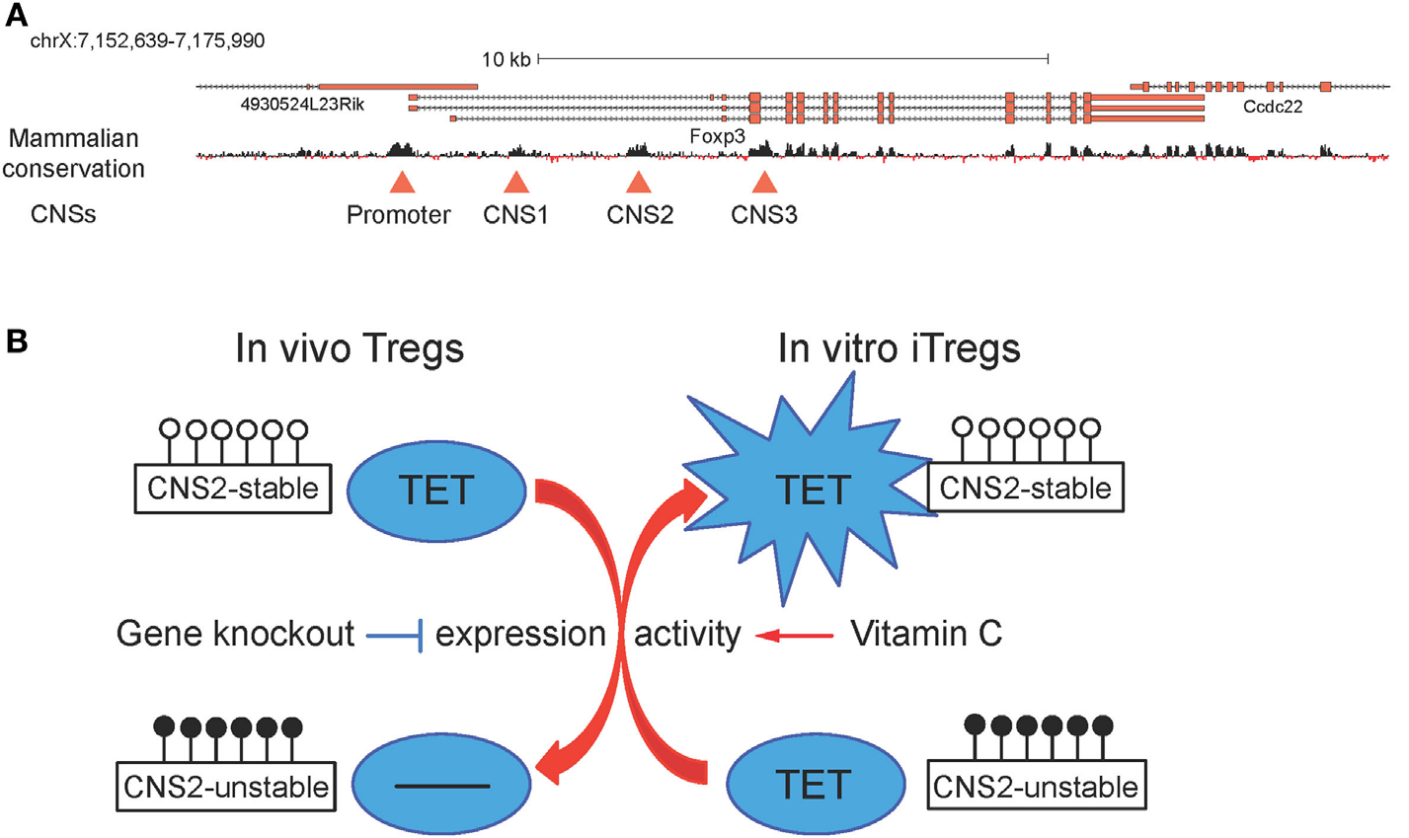
**(A)** Schematic representation of the *Foxp3* locus. *Foxp3* transcript variants and the mammalian conservation track are shown, and the conserved promoter region and the three conserved non-coding sequences (CNSs) corresponding to the intronic enhancers *CNS1, CNS2*, and *CNS3* are highlighted with red triangles (26). **(B)** Ten-eleven translocation (TET) proteins regulate *CNS2* methylation and T regulatory cells (Tregs) stability. *In vivo*, Tet deletion leads to increased DNA methylation in *CNS2* and impaired Treg stability; *in vitro*, addition of vitamin C during induced T regulatory cells (iTregs) generation boosts TET activity and results in decreased DNA methylation in *CNS2* and enhanced iTreg stability.

Mice with combined disruption of the *Tet2* and *Tet3* genes mediated by *CD4Cre* displayed unstable Foxp3 expression, concomitantly with DNA hypermethylation in *CNS1, CNS2*, and other TSDRs (Figure 7A) (26). Although Tet2 and Tet3 are the major TET proteins expressed in differentiated tissues and cell types, including lymph nodes and spleen (21), combined deletion of Tet1 and Tet2 also resulted in *CNS2* hypermethylation, impaired Treg cell differentiation and function, and autoimmune disease (102). This study also reported that hydrogen sulfide (H2S) was required for Treg cell differentiation and function by promoting the expression of Tet1 and Tet2, which were recruited to the Foxp3 locus by TGFβ-activated Smad3 and IL2-activated Stat5 to maintain *Foxp3 CNS2* demethylation and Treg-cell-associated immune homeostasis (102). It is likely that all three TET proteins play important roles in maintaining TSDR demethylation and Treg stability/function.

In a small molecule screen performed in mES cells, vitamin C was identified as a component of cell culture media required for germline gene induction and shown to act by increasing TET activity and consequently promoting DNA demethylation at germline gene promoters (123). Consistent with this report, vitamin C potentiated TET-mediated demethylation of TSDRs and increased the stability of Foxp3 expression in differentiating iTregs (Figure 7B) (26, 103). Administration of sulfinpyrazone, an inhibitor of a vitamin C transporter, confirmed that *CNS2* demethylation and the *in vivo* generation of peripheral Tregs were dependent on TETs and vitamin C (103). Vitamin C had the same effects on iTregs differentiated from human peripheral blood T cells; and both human and mouse iTregs generated in the presence of vitamin C showed suppressor activity comparable to that observed in Tregs isolated *ex vivo* (26). Collectively, these findings suggest that vitamin C and other small molecule activators of TET protein function might be valuable tools to stabilize *in vitro*-generated iTregs for clinical applications.

### B Cells

Similar to iNKT cells (67), pro-B cells preferentially express Tet2, Tet3, and, to a much lesser extent, Tet1. We and others have shown that Tet2 and Tet3 are required for early B cell development: deletion of both the Tet2 and Tet3 genes in early B cells with *Mb1Cre* resulted in a developmental arrest at the pro-B to pre-B stage, at least partly reflecting impaired Igκ rearrangement (46, 76). TET proteins regulate multiple aspects of B cell development at this stage, potentially by regulating the accessibility of key enhancers at the chromatin level (46). Our evidence is consistent with a model in which “pioneer” transcription factors such as PU.1 initially bind to enhancers with a “closed” chromatin conformation; subsequent recruitment of TET proteins to these regions followed by TET-dependent 5hmC deposition and/or DNA demethylation then facilitates chromatin remodeling at the Igκ and other enhancers required for Igκ rearrangement. In addition, TET proteins regulate the expression of IRF4/8, two transcription factors important for inducing Igκ rearrangement. Importantly, the observed phenotypic changes in Tet2/3-deficient B cells were reversible and dependent on TET catalytic activity, as re-expression of the active WT but not a mutant inactive TET2 catalytic domain partly restored Igκ enhancer accessibility and rearrangement (Figure 6).

## TET Loss-of-Function and Cancer

Both TET1 and TET2 are implicated in cancer: TET1 is an MLL partner in cases of acute myeloid and lymphoid leukemias (124) and has been reported to function as an oncogene in MLL-rearranged leukemias (125) as well as a tumor suppressor in other contexts [reviewed in Ref. (126, 127)]. In contrast, TET2 is clearly a tumor suppressor in all hematopoietic cell types reported so far, since deletions and loss-of-function mutations in the *TET2* gene are strongly associated with myelodysplastic syndromes, myeloproliferative neoplasms, and myeloid leukemias as well as lymphoid malignancies (126, 127). In both myeloid and lymphoid malignancies, *TET2* mutations occur in somatic cells, not in the germ line, and “second hit” mutations in other genes are typically required for progression to a fully malignant phenotype (126, 127). Frequent mutations in *TET2*, and sporadic mutations in *TET3*, have also been noted in peripheral T cell lymphoma (PTCL), including angioimmunoblastic T cell lymphoma and PTCL-NOS (PTCL, not otherwise specified) (128–130), and the malignant T cells often bear NK cell markers, thus resembling NKT cells (130). TET3 is rarely mutated in human cancers, but loss of TET function—as judged by low 5hmC levels—is frequently observed in many types of cancers as discussed below (126, 127).

### Diverse Human Cancers Show TET Loss-of-Function without TET Coding Region Mutations

A large fraction of myeloid malignancies without TET2 (or other TET) mutations displayed low 5hmC levels, pointing to profound loss of TET enzymatic activity (36, 127). Low 5hmC levels in the absence of *TET* coding region mutations have also been documented in breast cancer, melanoma, and other cancers (126, 127). The underlying mechanisms include decreased TET mRNA/protein expression due to promoter methylation, microRNA upregulation, or increased activity of E3 ubiquitin ligases; global inhibition of TET function, for instance by the “oncometabolite” 2-hydroxyglutarate produced by dominant recurrent mutations in *IDH1* and *IDH2*, predominantly in the context of glioblastoma and myeloid leukemias [reviewed in Ref. (131)]; or aberrant regulation of the nuclear-cytoplasmic localization of TET proteins (especially TET3) [reviewed in Ref. (127)]. Because TET proteins are utilizing molecular oxygen (132), 2-oxoglutarate (a product of the Krebs cycle) and reduced iron (Fe(II)) as substrates and co-factors (9, 10), TET enzymatic activity is likely to be sensitive to many signals including hypoxia, metabolic state (2-oxo-glutarate levels), Fe^2+^ availability, and the redox environment (127).

### TET Deficiency in T Cells Results in an Antigen-Driven, Transmissible iNKT Cell Lymphoma

As noted in the previous section, combined deficiency of Tet2 and Tet3 in T cells is associated with dramatic iNKT cell expansion as well as impaired T regulatory function (67). The iNKT expansion is driven by antigen recognition and not by Treg deficiency: transfer of small numbers of purified *Tet2/3 DKO* iNKT cells into healthy non-irradiated congenic recipient mice led to further expansion and the emergence of an iNKT cell lymphoma even in the presence of an intact immune system, whereas the cells did not expand upon transfer into similar recipient mice that lacked CD1d, the MHC Class I-like protein that presents lipid antigens to iNKT cells (67).

The iNKT cell lymphomas developing in *Tet2/3 DKO* mice display several features of the PTCL observed in *p53^−/−^* mice (133). Although most *p53^−/−^* mice develop thymic T cell lymphomas that may or may not spread to other organs, about 21% of these mice develop PTCL characterized by splenomegaly and hepatomegaly without thymic involvement. These *p53^−/−^* PTCL resemble the iNKT cell lymphomas of *Tet2/3 DKO* mice in many respects: staining with CD1d-αGalactosyl-Ceramide tetramers, expression of TCR chains bearing the invariant Vα14-Jα18 rearrangement, and upregulation of PLZF, LEF1, and Myc (67, 133). In both mouse strains, iNKT cell expansion driven by TCR stimulation was observed upon transfer of purified *Tet2/3 DKO* and *p53^−/−^* iNKT cells to WT but not CD1d-deficient mice (67, 133). Consistent with antigen-dependent expansion, development of iNKT cell PTCL in p53-deficient mice was accelerated by repeated injections with heat-killed bacteria expressing glycolipid antigens, and diminished by treatment of mice with anti-CD1d or cyclosporin A, a compound that interferes with TCR signaling by inhibiting the calcineurin/NFAT pathway (133). Connecting these observations, the myeloid leukemias developing after acute Tet2/3 deletion with Mx1Cre and polyI:polyC show impaired DNA repair (68); moreover, 5hmC was shown to be deposited at sites of DNA damage in x-irradiated HeLa cells (134). Whether and how cells with profound TET loss-of-function develop defects in p53-dependent or other DNA damage sensing pathways remains to be explored.

### TET Proteins and B Cell Malignancy

In addition to regulating B cell development, TET proteins are essential tumor suppressors in B cells. Although *TET2* deletions and loss-of-function mutations are more frequent in myeloid malignancies, they were observed in approximately 2% of various B cell malignancies, including a total 5.7% of patients diagnosed with diffuse large B cell lymphoma (135). Tet1-deficient mouse B cell progenitors showed increased self-renewal *in vitro* and the mice developed B cell lymphomas at an advanced age (18–24 months) (136). Consistent with these mouse genetic studies, the TET1 promoter was shown to be hypermethylated and TET1 expression was repressed in human patients with non-Hodgkin B cell lymphoma (136). Intriguingly, whereas Tet2-deficient mice develop myeloid leukemia, dual Tet1/Tet2 deficiency resulted instead in the development of B cell lymphoma with delayed disease progression (12–15 months) compared to Tet2 deficiency alone (113). As discussed above, Tet1 behaves as a weak tumor suppressor in B cells but may be weakly oncogenic in HSPC, where it promotes the development of myeloid leukemia in the absence of Tet2. An alternative hypothesis, not mutually exclusive, is that *TET2* mutations predispose to multiple types of hematopoietic cancers, with myeloid leukemia is the most prominent and rapidly occurring.

In our own studies, mice with combined early deletion of Tet2 and Tet3 in developing B cells using *Mb1-Cre* developed progressive B cell lymphoma and succumbed to disease within 5–6 months of age, an earlier onset compared to 15–20 months observed in Tet1/2-deficient mice (46, 113). TET3 mutations are rarely found in human hematological cancers (137), but nevertheless, these results clearly demonstrate the function of TET proteins in preventing B cell and other hematopoietic malignancies.

## Open Questions and Future Directions

Extensive research in immune cells and other cell types over the last several years has shed light on the *in vivo* functions of TET proteins, revealing a profound influence of these proteins on immune development and function (summarized in Table 1). However, many questions remain to be answered.

**Table 1:** **Summary of immune phenotypes in *Tet* mutant mice**.

T and B cell phenotypes in ten-eleven translocation (TET)-deficient mice		
Genotype	Phenotype	Reference
*Tet2*^fl/fl^ *Cd2Cre*: deletion of Tet2 in hematopoietic cells	– Reduced secretion of signature cytokines under *in vitro* polarization toward helper lineages – Reduced *in vivo* secretion of cytokines	Ichiyama et al. (45)

*Tet2*^−/−^: germline deletion of *Tet2*	– Normal T cell and B cell development	Ko et al. (110)

*Tet1*^−/−^: germline deletion of *Tet1*	– Enhanced stem cell proliferation – Pro-B-cells show enhanced DNA damage – B cell lymphocytosis	Cimmino et al. (136)

*Tet1*^fl//fl^ *Tet2*^fl//fl^ *Mx1Cre*: interferon-inducible deletion of *Tet1* and *Tet2* in hematopoietic cells	– B-ALL emergence	Zhao et al. (113)

*Tet2^+/^*^−^*Tet3*^fl//fl^ *CD4Cre* and *Tet2*^fl//fl^ *Tet3*^fl//fl^ *CD4Cre* mice: germline deletion of *Tet2* plus*Tet3* deletion in T cells (beginning at the DP thymocyte stage) or simultaneous deletion of *Tet2* and *Tet3* in T cells (beginning at the DP thymocyte stage)	– Death due to lymphoproliferative disease at 8 weeks old – Reduced number of peripheral T regulatory cells and decreased stability of Foxp3 expression due to increased methylation of the *CNS2* intronic enhancer of the *Foxp3* locus – Crucial role of vitamin C for TET activity – Invariant NKT cells (iNKT) cell lineage skewing and expansion – *Tet2/3* DKO iNKT cells can mediate a CD1d-restricted iNKT cell lymphoma	Yue et al. (26) Tsagaratou et al. (67)

*Tet2*^−/−^*Tet3*^fl//fl^ *Mb1Cre*: germline deletion of *Tet2* and deletion of *Tet3* early in the B cell lineage	– Blockage from pro-B to pre-B – Decreased number of CD19^+^B220^+^ B cells in spleen (8–11 weeks old) – Increased number of IgM^–^ IgD^–^ B cells in spleen (8–11 weeks old) – Myeloid expansion in spleen – B cell malignancy at 20 weeks	Lio et al. (46)

*Tet2*^fl//fl^ *Tet3*^fl//fl^ *Mb1Cre*: simultaneous deletion of *Tet2* and *Tet3* early in the B cell lineage (6- to 8-week-old mice)	– Blockage from pro-B to pre-B – Normal number of splenic follicular B cells with more proximal Vκ usage – Increased number of IgM^–^ IgD^–^ B cells in spleen – Decreased splenic marginal zone B cells and peritoneal B1 cells – Decreased T-dependent antibody response – Myeloid expansion in spleen	Orlanski et al. (76)

*Tet1*^−/−^ *Tet2*^fl//fl^ *CD4Cre*: deletion of *Tet1* in the whole organism plus deletion of *Tet2* in T cells (DP stage)	– Impaired Treg development and function	Yang et al. (102)

*Tet2*^fl//fl^ *LckCre* and *Tet2*^fl//fl^ *CD4Cre*: T cell-specific deletion of *Tet2*	– Impaired CNS2 demethylation of the *Foxp3* locus, role of vitamin C in promoting TET activity	Sasidharan Nair et al. (103)

Although the genomic distribution of 5hmC and other oxi-mC have been determined in different cell types, it has not yet been possible to define the genomic regions at which TET2 and TET3 exert their effects in each cell type of interest. This has primarily been due to the lack of commercially available antibodies that are suitable for chromatin immunoprecipitation. Thus, it has not been possible to distinguish overlapping versus non-overlapping functions of the TET2 and TET3 is cells lacking both proteins.

It is also not clear to what extent the catalytic activity of TET enzymes is needed for their diverse functions, as implied by the complex phenotypes of gene-disrupted mice. The interaction of TET1 with the co-repressor SIN3A in ES cells (138) and of TET2 with HDAC in macrophages (51) may confer functions that depend on protein–protein interactions and the stabilization of larger protein complexes rather than on TET catalytic activity. Further research is needed to shed light on these possibilities.

Another open question in the field is how TET proteins exert their tumor suppressive functions. Among the various possibilities are loss of proliferation control; dysregulated expression of cell cycle-related genes, tumor suppressors or oncogenes such as *Myc*; accumulation of DNA breaks; and loss of genomic integrity (46, 67, 68, 136). Unraveling which of these events is a direct consequence of TET loss and which is indirect will be a challenging task. To illustrate, although loss of TET function has been associated with increased DNA breaks in several systems (68, 134, 136), it is not yet established whether TET proteins directly participate in genome stability, for instance, by recruiting DNA repair factors via 5hmC recognition or through direct protein–protein interactions. Future mechanistic studies that incorporate a kinetic component will be needed to compare the relative time course of TET gene deletion and loss of 5hmC from the genome, with the time course of the diverse consequences of TET loss-of-function that have been observed in different cell types.

## Author Contributions

AT, C-WJL, XY, and AR set goals and wrote the manuscript.

## Conflict of Interest Statement

The authors declare that the research was conducted in the absence of any commercial or financial relationships that could be construed as a potential conflict of interest.
